# Innovation Management? Orienting Sepsis R&D and Technology Transfer Towards Stratified Medicine

**DOI:** 10.1016/j.ebiom.2016.04.007

**Published:** 2016-04-10

**Authors:** Vural Özdemir, Nezih Hekim

**Affiliations:** a TargeT Technology Transfer Office (TTO), Gaziantep University, Gaziantep 27300, Turkey; b Faculty of Communications and the Office of the President, International Technology and Innovation Policy, Gaziantep University, Gaziantep 27300, Turkey; c Amrita School of Biotechnology, Amrita Vishwa Vidyapeetham (Amrita University), Amritapuri, Clappana, P.O. Kollam, Kerala 690 525, India; d Department of Medical Biochemistry, School of Medicine, İstanbul Kemerburgaz University, Istanbul, Turkey

## 1 Innovating Sepsis R&D: The “Omics” Turn

The Third International Consensus Definitions Task Force has recently defined sepsis as a “life-threatening organ dysfunction due to a dysregulated host response to infection” (Singer et al., 2016). This broad framework is a clear advancement over multiple, inconsistent and narrow definitions employed in the past that tended to focus on infection or pro-inflammatory processes alone. Pro- and anti-inflammatory signaling occur simultaneously in host response to infection. Hence, we need in the current era a holistic and systems approach to decipher the large interindividual and between-population variability in pathogenesis of sepsis (Bauer et al., 2016, Wong et al., 2014).

Biomarkers that rapidly establish diagnosis and prognosis early in the course of sepsis are of particular interest for proactive interventions. Yet, the existing biomarker or diagnostic candidates fall quite short of the desired clinical sensitivity and specificity (Sims et al., 2016). There is no “typical” sepsis patient, nor a “universal therapy”, given that sepsis is a complex syndrome driven by host-environment interactions. Moreover, that many of the clinical trials aimed at novel sepsis diagnostics and therapeutics have failed is attributable, in part, to such hitherto unaccounted host, infectious agent and environmental heterogeneity enacting on each patient. Had we conducted clinical trials that were better characterized for patient-to-patient variations in molecular etiologies and host responses, we would have been perhaps better poised to diagnose and prognosticate sepsis and its divergent outcomes (Sims et al., 2016, Özdemir et al., 2015b).

**Fig. 1:**
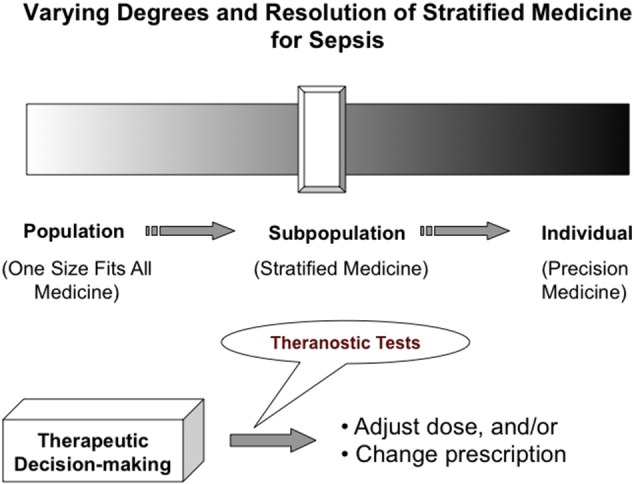
A strategic vision for rational diagnosis and prognostication of sepsis based on the varying degrees of stratified medicine.

Looking forward, Fig. 1 displays a vision of stratified medicine for sepsis whereby individuals at sepsis risk are characterized across multiple “omes” from genome to proteome to metabolome, thus facilitating multi-omics biomarker development (Higdon et al., 2015). Although achieving “high resolution therapeutics” for precision medicine at a single patient level might remain a long term objective, stratified medicine in cohorts of patients who share certain multi-omics biosignatures would improve the timeliness for establishing diagnosis and prognostication in sepsis. Theranostics, i.e., diagnostics that help forecast treatment outcomes, would be yet another deliverable and promise offered by multi-omics biomarkers and stratified medicine (Higdon et al., 2015, Özdemir and Kolker, 2016).

## 2 Transcriptomics and Metagenomics: New Frontiers

Using a sophisticated multiplexed transcriptomics and thre*e*-step biomarker development strategy in a German cohort, Bauer and colleagues report, in the present issue of *EBioMedicine*, on parallel occurrence of *both* pro- and anti-inflammatory changes in sepsis; they suggest a composite biomarker set that might be used in clinical utility studies in the future.(Bauer et al., 2016) This innovative study has confirmed their findings in an independent Greek cohort, a unique strength that makes the study more robust and generalizable as the Greek cohort is geographically and genetically different than the German cohort. The observations made by Bauer and colleagues lend support for the power of omics systems science research in sepsis. It is also a distinct foundational progress that can catalyze future multi-omics R&D integrating genomics, transcriptomics, proteomics and metabolomics research for sepsis diagnostics and theranostics.

We shall take this opportunity to underscore that host transcriptomic responses to sepsis, if coupled with tandem metagenomics characterization of the infectious pathogens, offer an unprecedented opportunity for innovation. Metagenomics concerns high-throughput, culture-independent, unbiased shotgun sequencing of DNA from environmental samples. Put in other words, metagenomics can help identify pathogens that escape the traditional culture-based methods in sepsis diagnosis and prognostication.

## 3 Bringing in the Environtome and Innovation Management

We think that the study by Bauer et al. (2016) signals the anticipated omics turn in the field of sepsis. Yet, we shall not forget to characterize the complex environmental factors that shape the multiple omes, including the transcriptome. In this context, we have recently defined the concept of *environtome* as “the entire complement of elements external to the human host, from microbiome, ambient temperature and weather conditions to government innovation policies, stock market dynamics, human values, political power and social norms that collectively shape the human host spatially and temporally” (Özdemir et al., 2015b). Sepsis R&D would thus be well served, no doubt, by a dual attention on multi-omics biomarkers as well as the sepsis environtome.

A related and rapidly emerging frontier is the new field of innovation management. Innovations, by definition, are new, different, or unprecedented, and they often result in a “rupture” with the past products, processes, and/or traditions. Indeed, it is an illuminating sign that we are dealing with a genuine innovation, if and when our past experience appears no longer adequate in our ability to respond and steer an alleged innovation.

Not surprisingly, innovation management and technology foresight research have become important topics and specialized professions over the past decade, as we currently witness a transition from material industries (e.g., textile, cement) to knowledge-based innovation (e.g., information technology, e-learning), requiring new skills for *knowledge management* worldwide (Birko et al., 2015).

Absent technology foresight and innovation management, new discoveries do not always come to fruition in the form of product or process innovations. Put in other words, there are many convincing examples and data that have taught us valuable lessons that *innovations and new technologies cannot be left alone*. Consider, for example, the health research and biotechnology innovation sector. Out of nearly US$ 240 billion spent annually on biomedical research globally, up to 85% is estimated as inefficient (Chalmers and Glasziou, 2009). One of the key reasons for such considerable research waste is poor steering or management of health research and innovation so it does not invariably lead to products or processes that have high relevance for the communities (e.g., patients, doctors, nurses, citizens) who meant to benefit from it.

As we gaze into the future, we shall also note, briefly, on innovation in research funding for sepsis R&D. Innovative funding designs on unresolved and yet shared health problems such as sepsis impacting the societies worldwide can lend themselves well to crowdfunding (Özdemir et al., 2015a). When coupled with sophisticated biological omics research as reported by Bauer et al. (2016) crowdfunding could open up new vistas for veritable funding of multi-omics research for discovery and replication in independent population samples worldwide.

Sepsis is a serious concern and yet the emerging exciting frontiers in biomedicine such as the study by Bauer et al. (2016) multi-omics (Özdemir et al., 2015b, Higdon et al., 2015, Özdemir and Kolker, 2016) and metagenomics technologies (Birko et al., 2015) and innovation foresight (Özdemir et al., 2015b, Özdemir and Kolker, 2016, Birko et al., 2015) offer us hope for veritable progress.

## Conflict of Interest Statement

We declare no competing interests.

## References

[bb0005] Bauer M., Giamarellos-Bourboulis E.J., Kortgen A. (2016). A transcriptomic biomarker to quantify systemic inflammation in sepsis — a prospective multicenter phase II diagnostic study. EBioMedicine.

[bb0010] Birko S., Dove E.S., Özdemir V. (2015). A Delphi technology foresight study: mapping social construction of scientific evidence on metagenomics tests for Water safety. PLoS One.

[bb0015] Chalmers I., Glasziou P. (2009). Avoidable waste in the production and reporting of research evidence. Lancet.

[bb0020] Higdon R., Earl R.K., Stanberry L. (2015). The promise of multi-omics and clinical data integration to identify and target personalized healthcare approaches in autism spectrum disorders. OMICS.

[bb0025] Özdemir V., Kolker E. (2016). Precision nutrition 4.0: a big data and ethics foresight analysis-convergence of agrigenomics, nutrigenomics, nutriproteomics, and nutrimetabolomics. OMICS.

[bb0030] Özdemir V., Faris J., Srivastava S. (2015). Crowdfunding 2.0: the next-generation philanthropy: a new approach for philanthropists and citizens to co-fund disruptive innovation in global health. EMBO Rep..

[bb0035] Özdemir V., Dove E.S., Gürsoy U.K. (2015). Personalized medicine beyond genomics: alternative futures in big data-proteomics, environtome and the social proteome. J. Neural Transm..

[bb0040] Sims C.R., Nguyen T.C., Mayeux P.R. (2016). Could biomarkers direct therapy for the septic patient?. J. Pharmacol. Exp. Ther..

[bb0045] Singer M., Deutschman C.S., Seymour C.W. (2016). The third international consensus definitions for sepsis and septic shock (sepsis-3). JAMA.

[bb0050] Wong H.R., Lindsell C.J., Pettilä V. (2014). A multibiomarker-based outcome risk stratification model for adult septic shock. Crit Care Med.

